# Cytogenetic screening of a Canadian swine breeding nucleus using a newly developed karyotyping method named oligo-banding

**DOI:** 10.1186/s12711-023-00819-w

**Published:** 2023-07-10

**Authors:** William Poisson, Alexandre Bastien, Isabelle Gilbert, Alexandra Carrier, Julien Prunier, Claude Robert

**Affiliations:** 1 Département des sciences animales, Faculté des sciences de l’agriculture et de l’alimentation, Université Laval, Québec, QC Canada; 2 Centre de recherche en reproduction, développement et santé intergénérationnelle, Québec, QC Canada; 3 Plateforme d’imagerie et microscopie, Institut de biologie intégrative et des systèmes, Université Laval, Québec, QC Canada; 4 Département de médecine moléculaire, Faculté de médecine, Université Laval, Québec, QC Canada

## Abstract

**Background:**

The frequency of chromosomal rearrangements in Canadian breeding boars has been estimated at 0.91 to 1.64%. These abnormalities are widely recognized as a potential cause of subfertility in livestock production. Since artificial insemination is practiced in almost all intensive pig production systems, the use of elite boars carrying cytogenetic defects that have an impact on fertility can lead to major economic losses. To avoid keeping subfertile boars in artificial insemination centres and spreading chromosomal defects within populations, cytogenetic screening of boars is crucial. Different techniques are used for this purpose, but several issues are frequently encountered, i.e. environmental factors can influence the quality of results, the lack of genomic information outputted by these techniques, and the need for prior cytogenetic skills. The aim of this study was to develop a new pig karyotyping method based on fluorescent banding patterns.

**Results:**

The use of 207,847 specific oligonucleotides generated 96 fluorescent bands that are distributed across the 18 autosomes and the sex chromosomes. Tested alongside conventional G-banding, this oligo-banding method allowed us to identify four chromosomal translocations and a rare unbalanced chromosomal rearrangement that was not detected by conventional banding. In addition, this method allowed us to investigate chromosomal imbalance in spermatozoa.

**Conclusions:**

The use of oligo-banding was found to be appropriate for detecting chromosomal aberrations in a Canadian pig nucleus and its convenient design and use make it an interesting tool for livestock karyotyping and cytogenetic studies.

**Supplementary Information:**

The online version contains supplementary material available at 10.1186/s12711-023-00819-w.

## Background

Cytogenetic analyses of domestic pigs have revealed more than 200 chromosomal rearrangements since the first investigations around 50 years ago [1]. Most of these are reciprocal translocations [2–4], whereby genetic material is exchanged between two chromosomes [2, 5]. In Canada, 0.91 to 1.64% of pigs carry a detectable chromosomal abnormality [6, 7], which is a lower rate than in Spain (3.8%) and Australia (6.8%) but higher than in France (0.5%) where systematic karyotyping of boars has been established since over 20 years [4, 8, 9]. Many chromosomal abnormalities are known to have an impact on fertility [6, 10, 11]. In cases of reciprocal translocation, litter sizes have been reduced by 17 to 100% with an average of 40% [6, 9, 12, 13]. In intensive hog raising, which relies heavily on artificial insemination, early detection is crucial, since a boar may produce over 3000 semen doses during its reproductive lifetime [14], leading to considerable economic losses due to recurrent reductions in litter size [6, 14, 15]. It has been estimated that each dollar invested in Canadian boar karyotyping yields an expected $5.30 return [7]. In addition to their impact on fertility, chromosomal abnormalities carried by boars that are used at insemination centres can be transmitted at varying frequencies to progeny [6, 9, 16–19], which lead to unchecked dissemination of defects, especially when progeny are selected within elite herds. Thus, the pattern of inheritance of rearrangements is of interest when carrier boars are considered valuable because of elite genetics or if the population is small.

Many methods to detect chromosomal abnormalities have been developed over the past few decades. The oldest are conventional chromosome banding techniques, namely Q-banding and G-banding [20, 21]. These methods have been used extensively for pig karyotyping, but their shortcomings have led to the increasing use of molecular alternatives. One of these is fluorescence in situ hybridization (FISH), which has been used with somatic cells and gametes, such as spermatozoa. Many FISH probes have been produced using vectors such as bacterial artificial clones (BACs) [22–24]. With improvements in massively parallel synthesis of oligonucleotides, it has become affordable and widely accessible to produce them in huge amounts [25–27]. The use of hundreds to thousands of short oligos as FISH probes provides repeatable, specific and cost-effective means of targeting specific chromosomal regions [26, 28]. Oligo designs also allow high analytical flexibility and can be adapted to many FISH experimental designs [26, 28, 29]. Although its effectiveness as a cytogenetics tool is now recognized in many fields [28, 30–34], including karyotyping [35], to date, this method has not been applied to pig.

In the present study, we present a new chromosome banding method for swine, developed by applying an existing oligo-based technology called OligoPaint FISH [26] and a defined probe structure [29] to karyotyping and sperm analysis. The resulting technology, called oligo-banding, provides a robust and cost-effective method to karyotype individuals, which is also technically simple and produces results that are easy to interpret with only little prior knowledge on cytogenetics.

## Methods

All chemicals were purchased from ThermoFisher (Mississauga, ON, Canada) unless specified otherwise.

### Oligonucleotide design

**Fig. 1:**
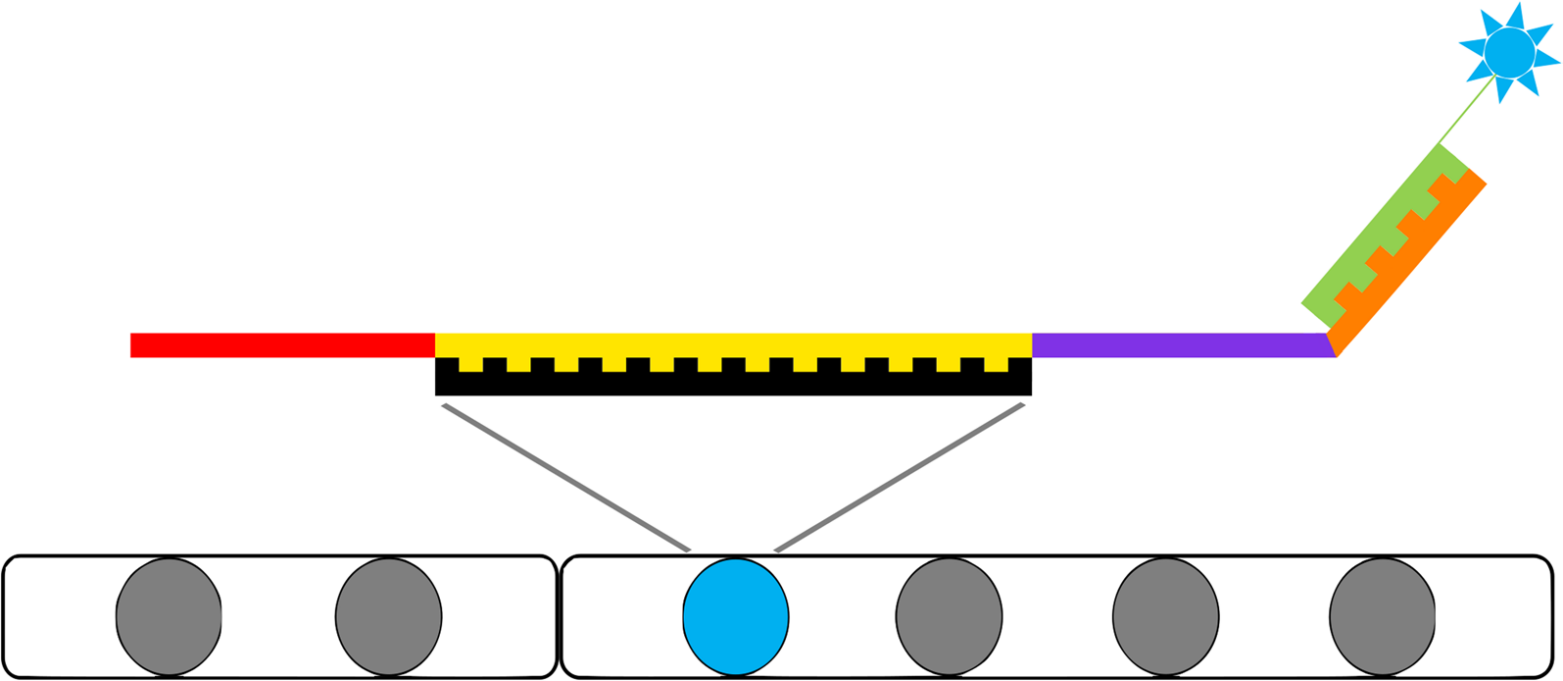
The four components of the oligonucleotide structure. Twenty-mer (red) sequence complementary to the reverse primer sequence; 39mer (yellow) sequence bound to a denatured segment of chromosomal DNA (black); 20mer (purple) sequence complementary to the forward primer sequence; 20mer (orange) sequence to which the detection oligo (green, 20mer) binds during the second hybridization step. Thus, the fluorophore (blue) colours the specific segment of the chromosomal DNA

Oligo structure has been described previously [29]. Briefly, four substructure sequences were joined: a genome-homologous 39mer, two 20mer orthogonal sequences for forward/reverse primer pairing and one for signal detection (complementary to the fluorophore handle) (Fig. 1). The sequences were determined as described previously [36] using OligoMiner [37], iFISH-probe-design (ifpd) [29] and orthogonal oligo design for FISH (OOD-FISH) [29]. Briefly, 39mer sequences were chosen by applying the OligoMiner tools in “balance” mining mode. A repeat masked Sscrofa11.1 assembly was used as starting material. *Blockparse.py, bowtie2, outputclean.py, kmerfilter.py* and *structureCheck.py* were respectively applied to select potential sequences, check for off-target sequences, process bowtie2 output files, and search for high-abundance kmers and secondary structures. OligoMiner output sequences were selected using Ifpd v2.0.4, of which the *Ifpd mkdb* and *ifpd dbchk* scripts were used to build reference databases and the *ifpd query set* script was used for proper sequence selection with the –n-oligo parameter set at 3000 oligos per probe. Within the proposed sets, probes were selected based on the distance between them and their distance from chromosome termini for clear detection signals. For probes labelled with one fluorophore only, three groups of 1500 oligos (start to middle, first quarter to third quarter, middle to end) were defined using an in-house *R* script, and the densest group was retained. For paired fluorophore probes, the 3000 oligos were retained (1500 per fluorophore, alternately).

Twenty-mer orthogonal sequences were obtained as described previously [36] based on a protocol described in Gelali et al. [29]. Briefly, six 20mer sequences were generated from each of the 240,000 25mer orthogonal sequences designed previously [38] and then used as input for the OOD-FISH pipeline [29]. The BLAST tool was used to search for alignments on the non-repeat masked Sscrofa11.1 reference genome, and sequences with an e-value > 25 were kept as candidates. Selection was based on self-dimerization and hetero-dimerization free energies with a threshold of − 9 kcal/mol. Final orthogonal 20mer sequence sets were chosen based on compatibility using PMC algorithms and attributed to the reverse or forward primer or to a “handle” sequence based on GC clamps [36].

The oligo substructure assembly (Fig. 1) was done using an in-house *R* script. The final 79mer sequences were purchased from Genscript (Piscataway, NJ, USA) (See Additional file 1: Table S1). Primers and labelled detection oligos were purchased from Integrated DNA technologies (IDT) as standard desalted or HPLC purified oligos (See Additional file 2: Table S2).

### Number of probes and colour attribution

**Fig. 2:**
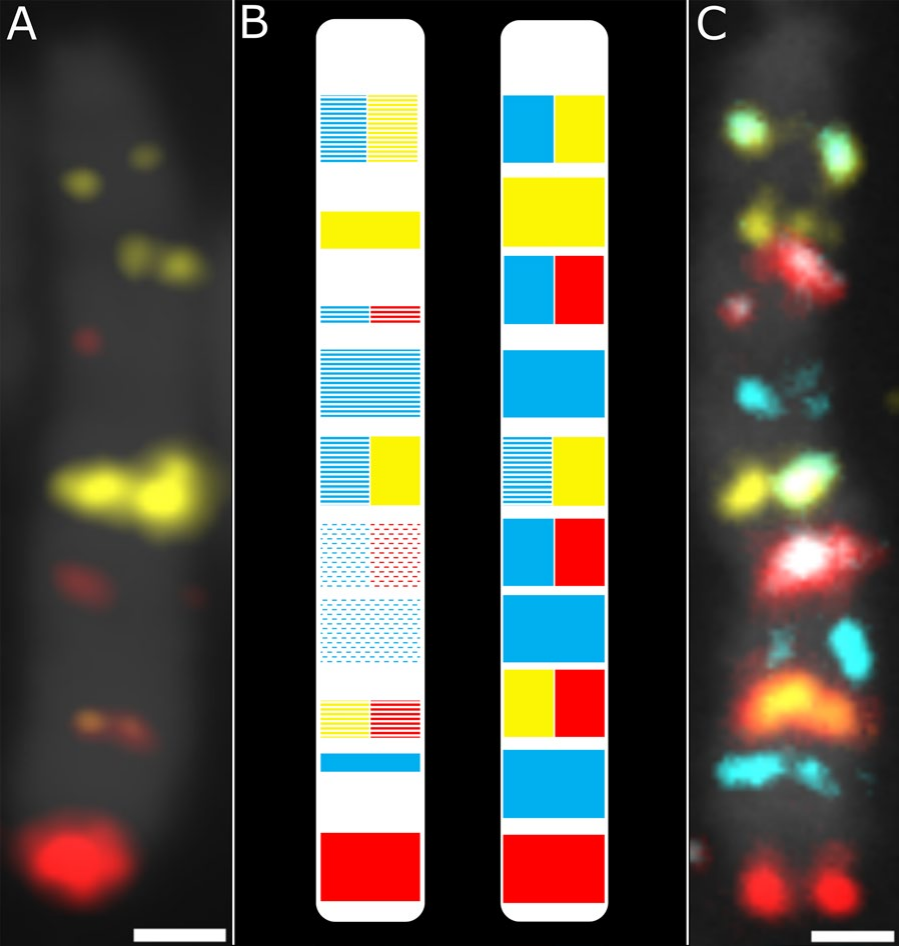
Determination of the sufficient number of oligonucleotides and oligonucleotide density and genomic coverage. **a** Hybridization of the test oligo pool on SSC13 showing insufficient signal intensity when oligo density or probe coverage (band widths) is reduced (the ATTO 425 signal merged with the background signal making it undetectable); and **b** schematic representations of test results (left) and optimized probe design (right). Dashes indicate minimal oligo density, lines indicate intermediate density, and solid colour indicates full density. Colour height (quarter, half, or full) represents genomic coverage. **c** final oligo-banding pattern of SSC13 showing strong signals from fluorophores 6-FAM, ATTO 550, and ATTO 647N. Oligo density and coverage were sufficient for the detection of chromosomal rearrangements. Probes 4 and 5 appear as a single band in this image because they were designed very close to each other. Scale bar = 1 µm

The number of probes and probe oligo density were determined in previous density and coverage experiments, in which 12,472 oligos were distributed differently among 11 probes on *Sus scrofa* chromosome 13 (SSC13) and two probes on SSCY (Fig. 2). Probe sets were then designed according to the best density and coverage obtained, to the distance between probes and the distance from chromosomal termini to optimize signal detection. For the 18 remaining chromosomes plus the completion of the previous 11 SSC13 test probes, 195,375 oligos were distributed among 84 probes. To maximize colour pattern differences between chromosomes, a *random banding* in-house Matlab script was used to generate 1000 sets of karyotype colour patterns and select the best set based on Levenshtein distance [39]. Colours were obtained by using fluorophores singly (i.e. 6-FAM, ATTO 550 or ATTO 647N) or in pairs (i.e. 6-FAM/ATTO 550, 6-FAM/ATTO 647N, ATTO 550/ATTO 647N) to generate six colours for karyotype patterning.

### Probe synthesis

Probe synthesis was a modified version [36] of a previous method [40]. Briefly, real-time PCR with PerfeCTa SYBR Green Fastmix (Quantabio, Beverly, MA, USA) in a LightCycler 480 II (Roche, Rotkreuz, Switzerland) was done for oligo pool amplifications and 5’ T7 RNA polymerase binding site and 3’ adapter sequence for oligo detection insertions. PCR products were purified using SparQ PureMag beads (Quantabio, Beverly, MA, USA). Specific complementary RNA was then transcribed in vitro using a HiScribe T7 High Yield RNA Synthesis Kit (New England BioLabs, Ipswich, MA, USA) and purified using RNA Clean XP (Beckman Coulter, Mississauga, ON, Canada). Finally, purified RNA was reverse-transcribed using Maxima H Minus Reverse Transcriptase, and cDNA was purified using Zymo-Spin IC Columns (Cedarlane, Burlington, ON, Canada). NanoDrop One (ThermoFisher Scientific, ON, Canada) was used according to the manufacturer’s instructions to quantify the polynucleotides after each purification step. Since oligos contained a chromosome-specific reverse primer region, probes were synthesized in chromosome sets to allow chromosome-specific hybridization, if needed.

### Lymphocyte culture and sample preparation

Blood samples from 34 normal or potentially translocated boars (Duroc, Yorkshire and Landrace) of a commercial swine nucleus breeding herd were taken by experienced staff who applied procedures that comply with national animal care standards. All the animals from this nucleus had been G-band karyotyped previously by a commercial karyotyping service provider and animals with an abnormal karyotyping report between 2019 and 2021 were tested with the current method. Chromosomal abnormalities were not revealed by the commercial partner before oligo-banding testing in order to test the ability to find them blindly. Normal boars were selected randomly. Lymphocytes were cultured in “PB-MAX” medium according to the manufacturer’s instructions [41] with slight modifications. Briefly, 0.5 mL of heparinized peripheral blood was suspended in 10 mL of medium and cultured for 48 h at 37 °C under 5% CO_2_. Metaphase arrest was induced by incubation in 0.5 µg/mL of KaryoMAX colcemid solution for 2 h, then suspending the cells in KaryoMAX hypotonic potassium chloride (0.075 M) for 12 min at 37 °C. Fresh ice-cold Carnoy’s fixative (methanol:acetic acid 3:1) was then added drop-by-drop and the suspension was left on ice for 10 min. The fixing procedure was repeated once. The fixed cell suspension was dropped onto slides from a height of 6 inches at room temperature and 55% humidity. The slides were aged for 24 h at room temperature. Samples were denatured with 70% formamide/2X saline-sodium citrate (SSC) for 2 min at 70 °C and then quenched and dehydrated in 70, 90 then 100% ethanol for 3 min each at − 20 °C. Slides were air-dried prior to hybridization.

### Hybridization of oligo-banding probes

The hybridization steps have been described previously [36]. Probes were hybridized at 0.4 µM in 50% formamide and 10% dextran sulfate in 2X SSC solution covered with a coverslip for 16 to 18 h using an Array Booster AB410 humidified chamber (Advalytix AG, Brunnthal, Germany) at 40 °C. The slides were washed four times by immersing in 2X SSC + 0.1% Tween 20 (SSCT), first for a quick dip to remove coverslips, then 15 min at 60 °C and twice in a fresh solution for 5 min at room temperature. Slides were air-dried prior to the next steps. To detect hybridization, 40 µL of detection oligo hybridization mixture (3 µM of each labelled detection oligo in 2X SSC/30% formamide) was spotted on the slide for 1 h followed by three washes in 2X SSCT (dipping, immersing for 10 min then 2 min), then immersing the slide in 0.2X SSC for 5 min (all steps at room temperature). Excess buffer was removed and True View autofluorescence was applied according to the manufacturer’s instructions (Vector Laboratories, Burlingame, CA, USA). Samples were counterstained and mounted in Vectashield Vibrance antifade medium with DAPI (Vector Laboratories) according to the manufacturer’s instructions.

### SpermFISH

Fresh semen (commercial, frozen at − 20 °C in 90% foetal bovine serum with 3% glycerol after centrifugation for 5 min at 2000 × *g*) was thawed and washed twice in phosphate buffered saline (PBS) solution, diluted 1/75 in PBS then smeared on slides as described by Hassanane et al. [42] with minor modifications. Smears were kept overnight in anhydrous ethanol at room temperature. Papain solution (1.25 mg + 0.155 mg dithiothreitol in 100 µL of 0.2 M Tris buffer at pH 8.6) was applied for decondensation, followed by dipping the slides twice in 0.2 M Tris buffer at room temperature. Slides were immersed in three changes of anhydrous ethanol, then air-dried, immersed overnight in Carnoy’s fixative at − 20 °C, air-dried and dehydrated in 70, 90 then 100% ethanol each for 3 min at room temperature and then denatured as for chromosome spreads. Hybridization was performed as described above.

### Imaging and analysis

A Nikon Eclipse e600 microscope was used with the 100X/1.4NA oil immersion objective and C-SHG1 super-high-pressure mercury lamp. Filters were purchased from Nikon or Chroma. Fluorescence was detected under the conditions summarized in Table 1. Images were acquired using a QImaging EXi Blue CCD Camera with QCapture Pro 7 software. Oligo banding in-house ImageJ plugin [39] was used to visualize the expected banding pattern and to detect chromosomal abnormalities based on automatic banding pattern analysis (see Additional file 3: Fig. S1, Additional file 4: Fig. S2, Additional file 5: Fig. S3). ImageJ was used to generate all graphic illustrations except for Fig. 1 (Inkscape) and Fig. 4 (Paint 3D).

**Table 1:** Fluorophores used for probe detection

Fluorophore	Excitation wavelength (nm)	Emission wavelength (nm)
DAPI	340–380	435–485
6-FAM	465–495	515–555
ATTO 550	510–560	> 570
ATTO 647N	625–655	665–715

## Results

### Probe design

**Fig. 3:**
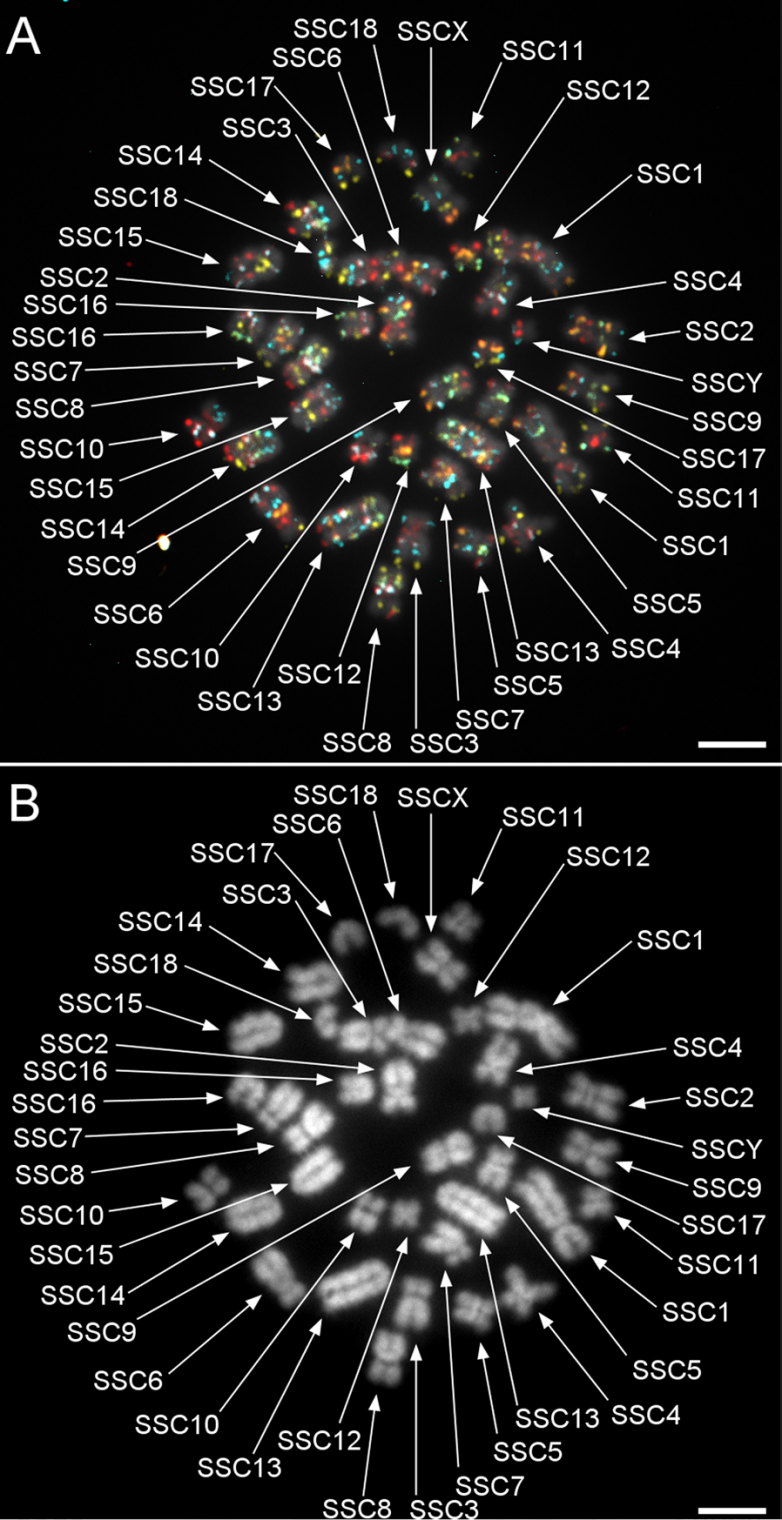
Determination of centromere positions. **a** Hybridization of all probes on a late metaphase lymphocyte spread (on which chromatid attachment points are easier to distinguish); and **b** using the DAPI channel as reference to highlight centromeres. Scale bars = 5 µm

Based on published studies and preliminary experiments, and using publicly available tools, 207,847 oligos (79mer) were distributed over 97 probes (1500 or 3000 oligos per probe) in this study (See Additional file 1: Table S1). The number of probes per chromosome ranged from 2 (Y chromosome) to 11 (for SSC1 and SSC13, the two largest chromosomes). Probe distribution over the genome (Table 2) revealed an average genomic distance between two neighbouring probes of 28.45 Mb (18.57–35.41 Mb, SD 4.18 Mb) and a mean distance of the first and last probes from the chromosome termini of 6.79 Mb (6.26–9.55 Mb, SD 0.85 Mb). Preliminary experiments on SSC13 indicated, via the number of oligos, oligo density and genomic coverage, that maximal density (around 6.5 oligo/kb) and 1500 oligo per single colour band were optimal to provide sufficient fluorescence intensity for unambiguous detection (Fig. 2). These three parameters were used to generate the final probe sets. Overall, the probes covered a genomic region of 458.56 kb on average (317.05–567.71 kb, SD 61.35 kb). An average oligo density of 6.87 oligo/kb was generated (3.02–8.62 oligo/kb, SD 1.17 oligo/kb, Table 2). Preliminary experiments also showed that fluorophore ATTO 488 yielded poor results, since the counterstain (DAPI) bled into its spectrum. Furthermore, removal of fluorescence bleeding reduced the ATTO 488 signals (Fig. 2a). This fluorophore was therefore replaced by FAM, which gave clearer signals (Fig. 2b). The final probes were hybridized successfully on chromosomes and showed clear and bright signals that allowed unambiguous identification of each chromosome (Fig. 3a). Since the oligos contained a chromosome-specific reverse primer region, the probes could be synthesized and hybridized in chromosome subsets.

**Table 2:** Parameters of the chromosome probe sets

SSC	Number of probes	Mean density (oligo/kb)	Mean coverage (kb)	Mean interprobe distance (Mb)	Mean distance from ends (Mb)
1	11	7.95	407.14	25.62	6.83
2	6	7.02	472.20	27.13	6.73
3	5	6.51	489.77	29.35	6.50
4	5	7.51	435.29	28.82	6.74
5	4	5.54	567.71	29.91	6.26
6	6	7.83	417.29	30.99	6.70
7	5	7.47	422.50	26.61	6.64
8	5	6.50	529.61	30.93	6.29
9	5	6.91	462.63	31.04	6.52
10	3	8.62	392.15	27.68	6.41
11	3	6.84	467.41	32.60	6.28
12	3	6.07	529.09	23.61	6.39
13	11	7.38	317.05	18.57	9.55
14	5	7.24	421.43	31.54	6.74
15	5	7.47	440.67	31.21	6.68
16	3	7.63	410.37	32.91	6.45
17	3	7.50	421.36	24.78	6.33
18	3	6.09	524.97	20.90	6.31
X	4	6.34	517.71	35.41	8.82
Y	2	3.02	524.80	29.41	6.55
Total	97	6.87	458.56	28.45	6.79

### Centromere positioning and G-banding equivalences

**Fig. 4:**
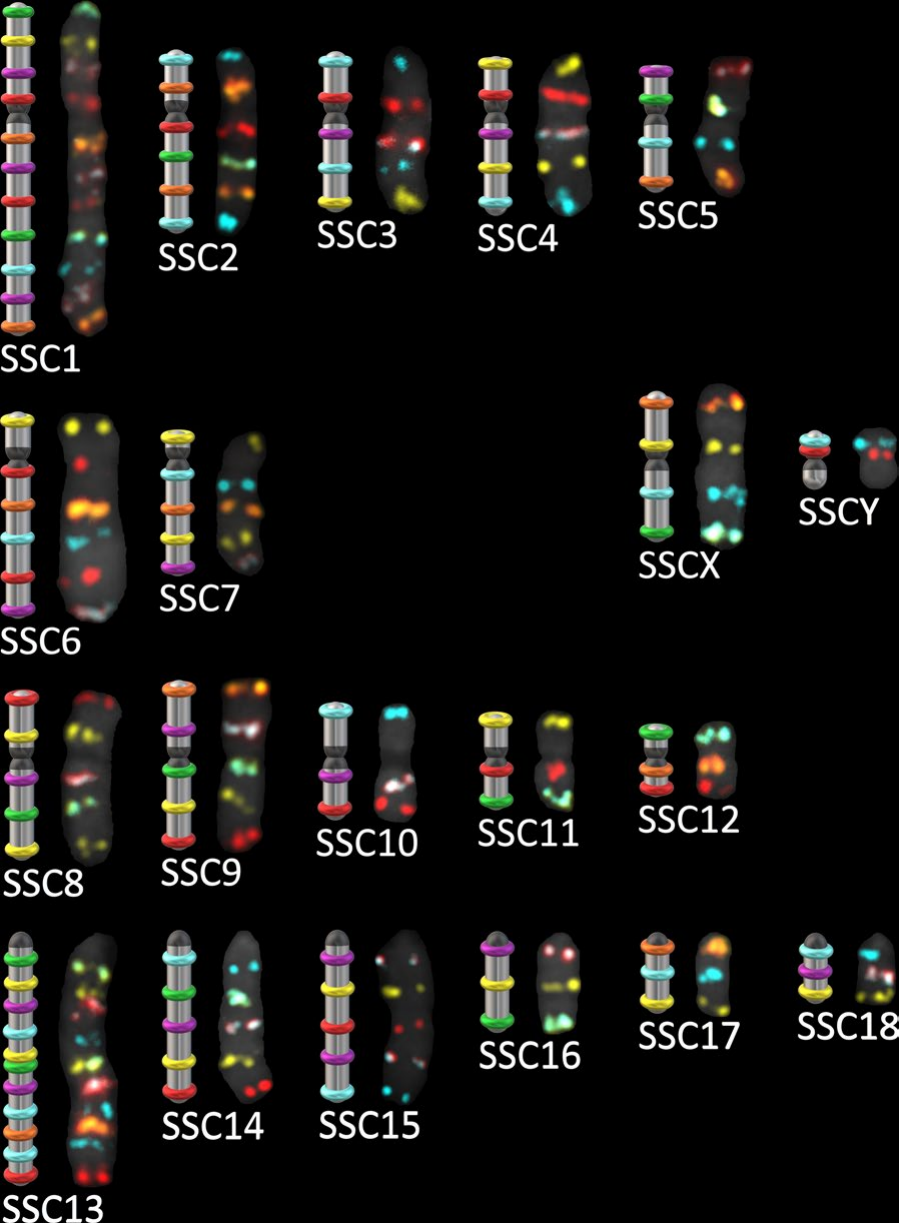
Graphic representation of a karyotype determined by oligo-banding. The colour scheme was determined to maximize the Levenshtein distance between chromosomes to ensure low colour repetition and unambiguous chromosome identification. Hybridized probes gave clear signals that match the graphic patterns. The centromere positions (black junctions or termini) were determined de visu based on the highlighted attachment point in the counterstain (DAPI) channel of labelled chromosomes

G-banding is the most used conventional method to detect pig chromosomal rearrangements. Thus, we determined equivalences between bands from G-banding and oligo-banding. Oligo-banding centromere positions were also determined to validate the equivalences. Centromeres were identified on chromosomal spreads that showed a high level of condensation. An optimal spread on which each chromosome was identified is shown in Fig. 3a. Comparison between fluorescence channels and counterstaining (Fig. 3b) allowed centromere positioning. The new fluorescent karyotype (Fig. 4) was based on previously established standards for chromosomal ordering [43]. G-banding equivalences were determined by comparing probe genomic addresses and G-band estimated positions according to Donaldson et al. [1] (See Additional file 6: Table S3). Differences in centromere positions between G-banding and oligo-banding were observed for SSC6, SSC7 and SSCY. For these chromosomes, G-band equivalences were attributed based on inverted DAPI-banding observed in this study instead of the estimated genomic position to conserve our centromere positions. Based on equivalences, 51.55% of the oligo-banding was within a light G-band, 44.33% corresponded to a dark band and 4.12% straddled the two banding types.

### Detection of chromosomal rearrangements

**Fig. 5:**
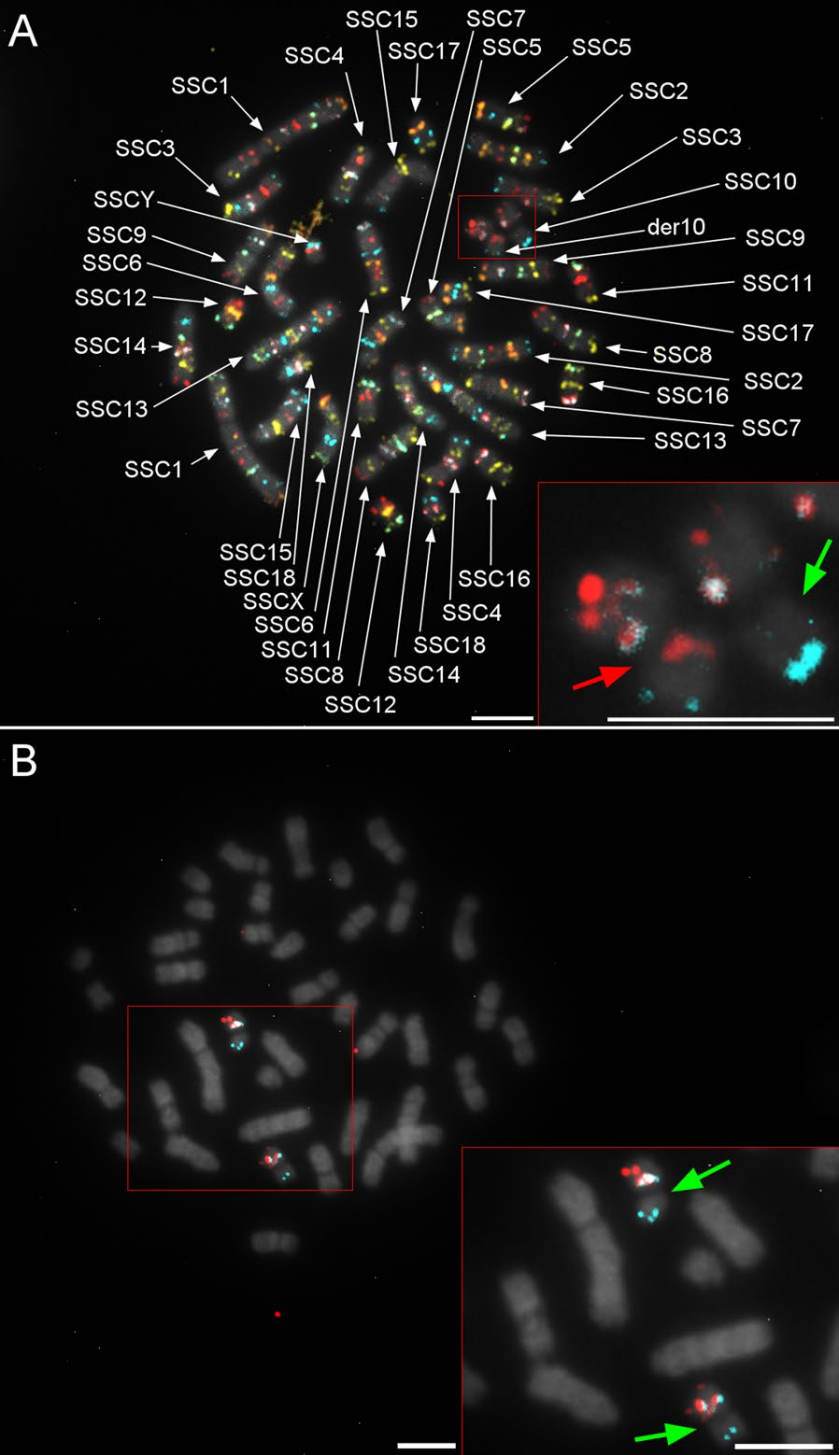
The add(10)(pter- > p11::?::p10- > qter) rearrangement carried by a heterozygous boar, detected using oligo-banding probes. **a** First hybridization with all probes to identify chromosomes and locate potential rearrangements; red labelling (red arrow) on one chromosome 10 homolog is derivative material (green arrow is normal material); and **b** confirmation of the unbalanced structural rearrangement on chromosome 10; hybridization of only the SSC10 probe set confirmed the addition of material from another chromosome, since the additional material is not labelled (green arrows). Scale bars = 5 µm

To detect chromosomal abnormalities, all probes were hybridized simultaneously on lymphocyte spreads to survey all chromosomes (Fig. 5a). Any deviation from expected patterns was considered a potential rearrangement (fluorescence artefacts, missing or duplicated signals, probe translocation or inversion, etc.). By analyzing the most intact chromosome spreads (5 to 10), it was possible to eliminate potential artefacts (chromosome piling, twisting, or folding) and determine the need for validation. Potential rearrangements were confirmed by hybridizing the probe subsets of the chromosomes involved in suspected abnormalities (Fig. 5b). Overall, both the conventional method and oligo-banding were able to detect four translocations. However, based on the previous G-banding karyotype report, the conventional method failed to detect the addition of material within one chromosome of a carrier boar. G-banding breakpoints were determined by comparing probe positions, G-banding equivalences and the inverted DAPI-banding pattern generated by the Vectashield counterstain.

#### Case I: add(10)(pter- > p11::?::p10- > qter) carrier

This unbalanced abnormality that consists in the addition of chromosomal material on one SSC10 homolog, between the first and second probes, was observed in each analyzed spread of the Duroc individual (Fig. 5a). This insertion generated a red band on the p arm near the centromere, which was not detected by conventional G-banding. SSC10 was the only chromosome that showed an abnormal banding pattern. Hybridization of the SSC10 probe subset confirmed that the additional material came from another chromosome, because of the absence of the supplementary red spot when probes of other chromosomes were not hybridized (Fig. 5b). The origin of the additional material could not be investigated further, since the boar was culled very soon after failing the semen conservation tests.

#### Case 2: rcp(1;7)(q15;q10) carrier

**Fig. 6:**
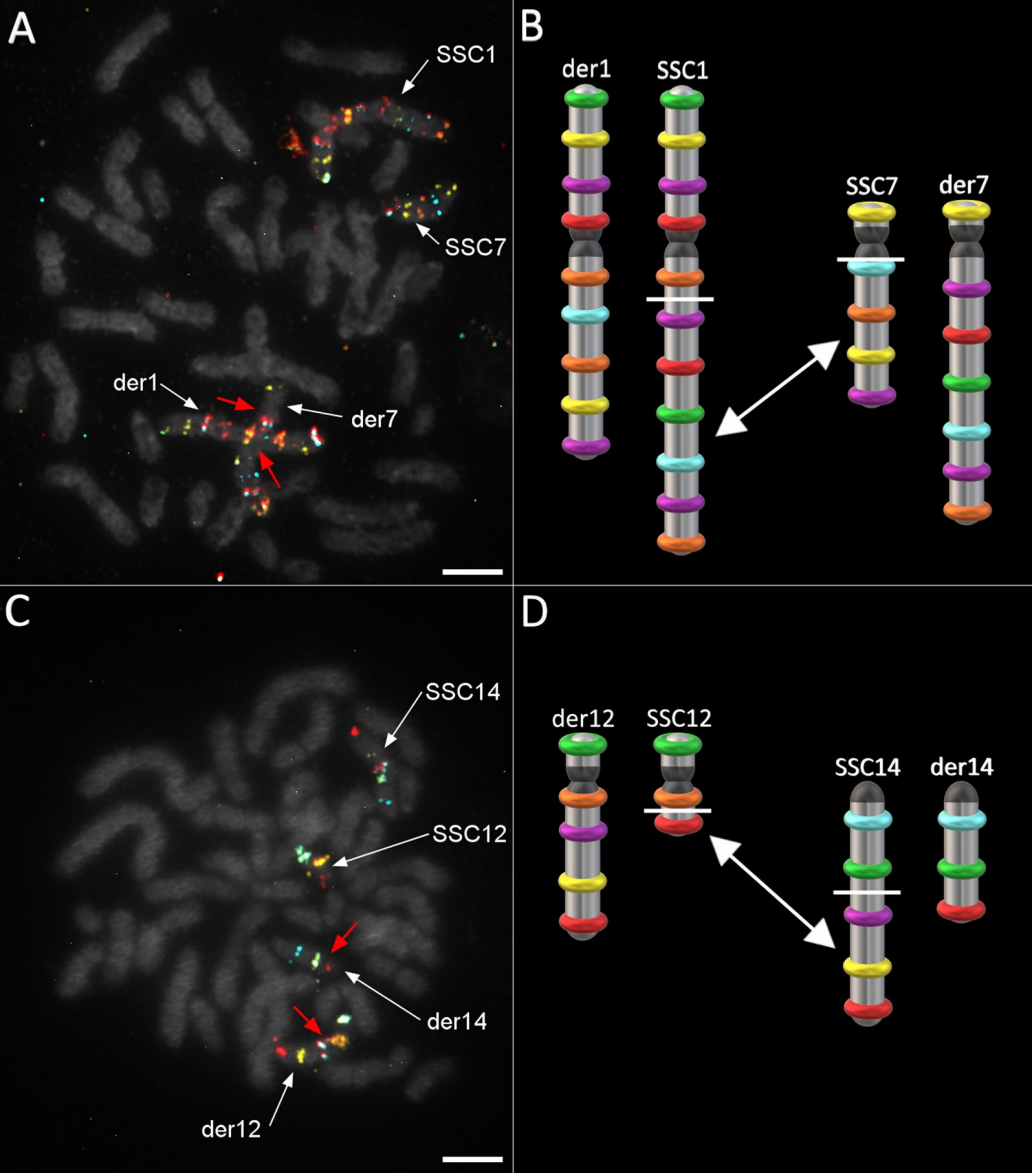
Detection and confirmation of rcp(1;7)(q15;q10) and rcp(12;14)(q13;q21) carrier boars. **a** Confirmation of the reciprocal translocation between SSC1 and SSC7 using their respective oligo-banding probes; **b** illustration of rcp(1;7) breakpoints (white bars) and translocations (arrow); **c** confirmation of SSC12/SSC14 translocation using the corresponding probes; and **d** illustration of the t(12;14) breakpoints (white bars) and translocations (arrow). Scale bars = 5 µm

This rearrangement implied the translocation of large fragments between SSC1 and SSC7 (Fig. 6a). The breakpoints were mapped between the fifth and sixth probes (from the top) on SSC1 and just beyond the SSC7 centromere, before the second probe (Fig. 6b).

#### Case 3: rcp(12;14)(q13;q21) carrier

Reciprocal translocations between SSC12 and SSC14 are one of the most frequently observed rearrangements among the commercial nucleus herd based on previous G-banding testing. In the present study, the translocation involved a small part of SSC12q bearing the third probe, and the portion of SSC14 between its second and third probe locations (Fig. 6c, d). Rcp(12;14) was the only translocation found in this study that involved an acrocentric chromosome.

#### Case 4: rcp(3;6)(q25;q21) Yorkshire carrier

**Fig. 7:**
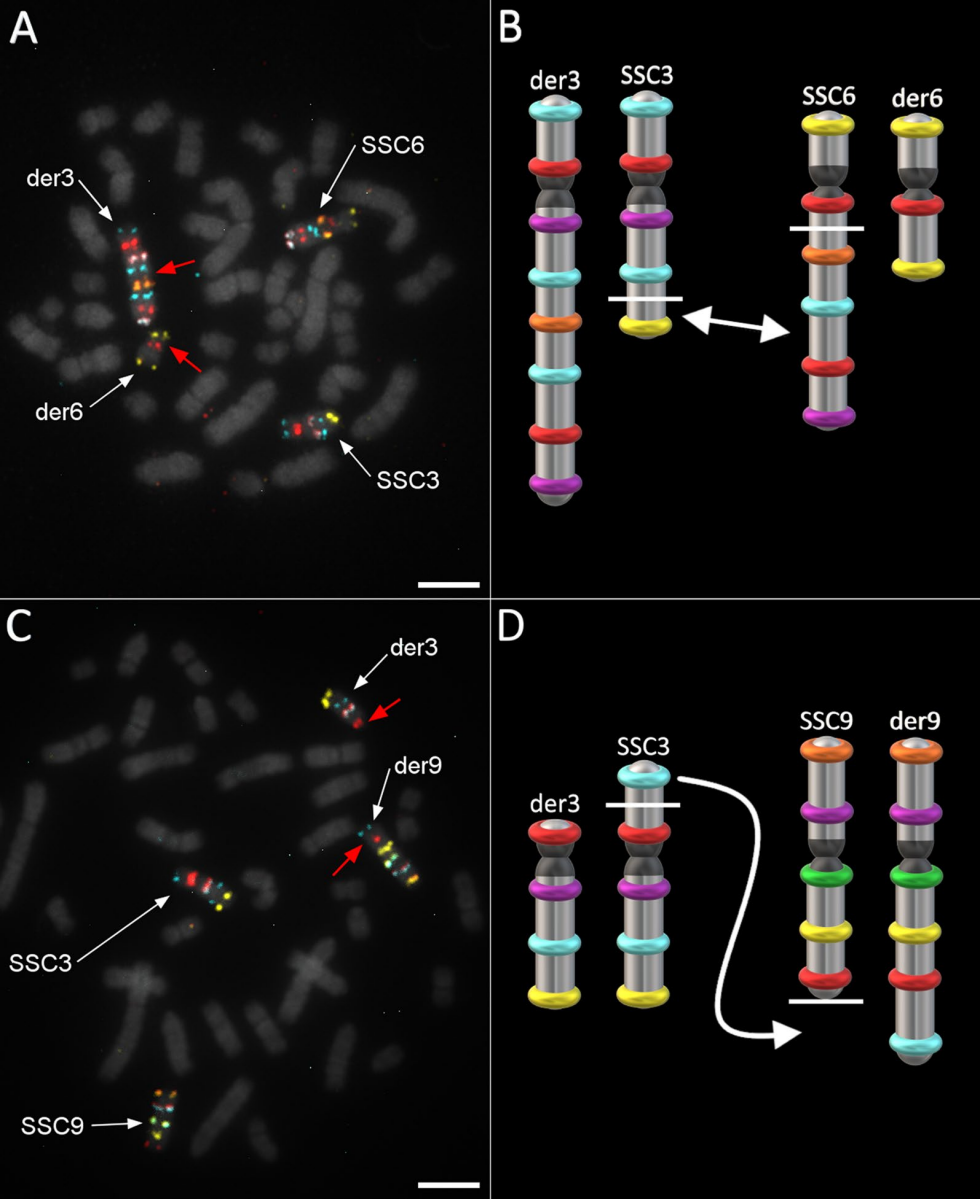
Detection and confirmation of rcp(3;6)(q25;q21) and t(3;9)(p13;q26) carrier boars. **a** Confirmation of the reciprocal translocation between SSC3 and SSC6 using their respective oligo-banding probes; **b** illustration of rcp(3;6) breakpoints (white bars) and translocations (arrow); **c** apparent SSC3;SSC9 translocation revealed by the corresponding probes (reciprocity not confirmed); and **d** illustration of the t(3;9) breakpoints (white bars) and translocations (arrow). Scale bars = 5 µm

This translocation occurred between a near terminal SSC3 fragment bearing the last probe and a larger SSC6 fragment bearing the four last probes (Fig. 7a, b). Thus, the SSC6 breakage occurred on its q arm. This rearrangement had no impact on spermatic parameters (73.19 × 10^9^ spermatozoa per ejaculate, 98.3% morphologically normal, 89.4% motile, which were measured prior to the cull).

#### Case 5: t(3;9)(p13;q26) carrier

The distal part of the SSC3 p arm bearing the first probe was translocated to the q terminus of SSC9 (Fig. 7c, d). Since this translocation was beyond the most distal probe on SSC9, it was not possible to determine from the oligo-banding patterns if the rearrangement was reciprocal. The absence of any visible unlabelled fragment at the top end of the derivative chromosome 3 (der3) suggests a translocation without reciprocal exchange of material with SSC9.

### SpermFISH

**Fig. 8:**
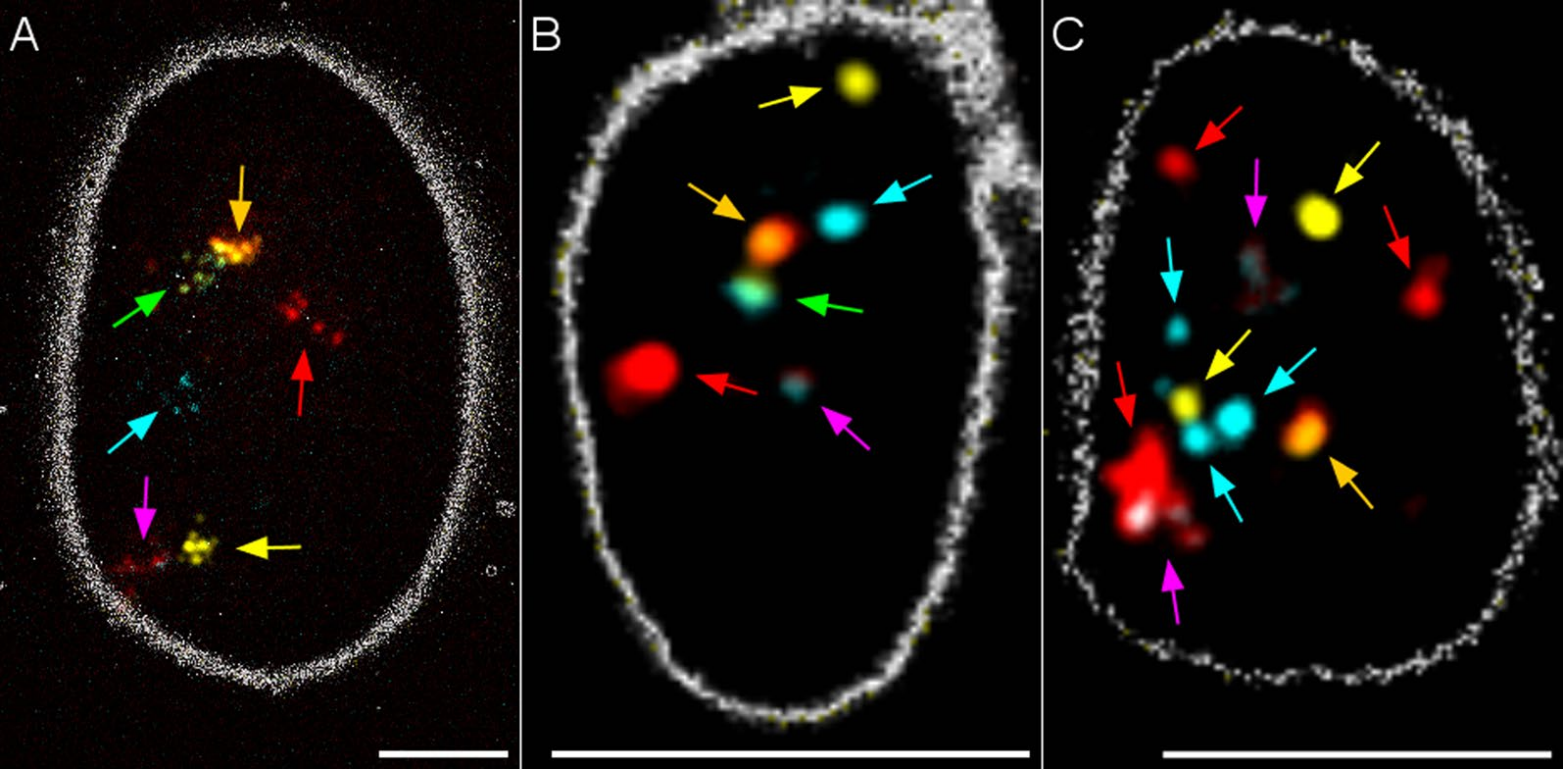
Results of SpermFISH using selected oligo-banding probes on boar spermatozoa. **a** Hybridization of SSC12 and SSC18 probes on normal boar spermatozoa; over-denaturation of DNA led to multiplication of probe signals; **b** hybridization on less decondensed spermatozoa heads gave one signal per probe (more certain identification); and **c** hybridization of SSC3 and SSC6 probes on a balanced spermatozoon from the rcp(3;6) carrier. The expected multiple signals tended to stack, complicating probe interpretation. Head edges are highlighted with Edges lookup table. Scale bars = 5 µm

**Fig. 9:**
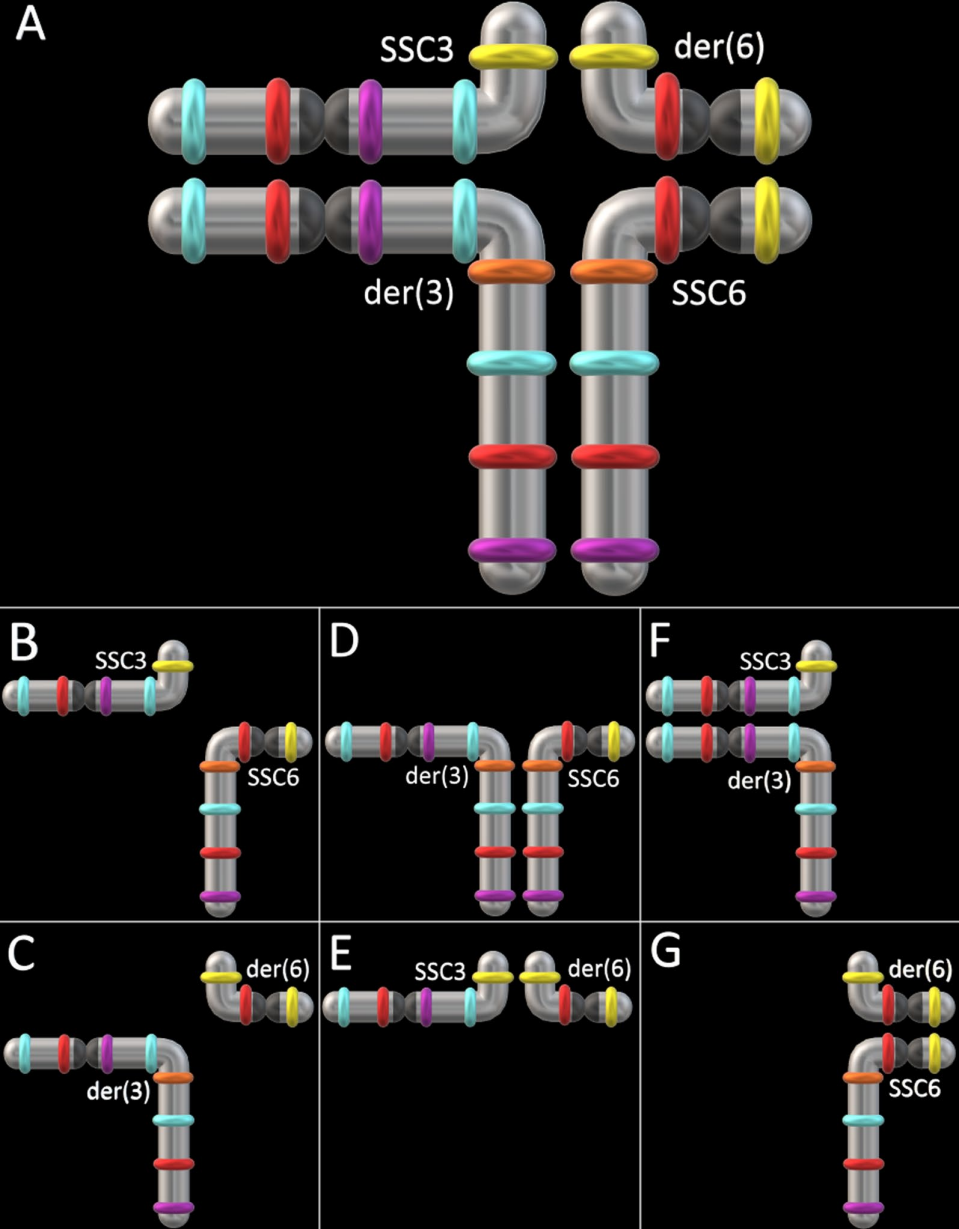
Graphic representation of 2:2 segregation types during meiosis in a rcp(3;6) individual. **a** Material exchange in a reciprocal translocation resulting in an expected quadrivalent pairing. Alternate segregation (the usual type of 2:2 segregation) results in balanced gametes with normal chromosomes (**b**) or derivative chromosomes (**c**); and adjacent-I segregation leads to unbalanced gametes with one derivative chromosome and the normal chromosome of the other pair (**d** and **e**). Adjacent-II segregation leads to unbalanced gametes with two copies of the same chromosome (one normal and one derivative) and no copy of the other chromosome (**f** and **g**)

Oligo-banding was tested as a means of detecting chromosomal abnormalities using spermatozoa heads from ejaculates. We began by hybridizing the six SSC12 and SSC18 probes on normal boar spermatozoa since these probes are each labelled with a different colour (Fig. 4). Each colour was detected. Less decondensed heads provided cleaner results with less signal multiplication (Fig. 8a, b). A decondensed head width of about 5 µm appeared to be optimal. SSC3 and SSC6 oligo-banding probes were then hybridized on spermatozoa from the rcp(3;6)(q25;q21) carrier. Diverse segregation types (alternate, adjacent I and II) were observed among labelled spermatozoa. Figure 8c shows the colour patterns that might be associated with a balanced karyotype (normal or reciprocally translocated) based on segregation patterns (Fig. 9).

## Discussion

Compared to BAC fragments, oligonucleotide-based FISH probes offer the advantage of being designed from a reference genome, which avoids the presence of repeated elements. Thus, the FISH probes designed are highly specific for any given genomic target, and the resulting band position patterns allow mapping of chromosomal breakpoints and facilitate further investigation of known genes within the genomic window. However, a common cytogenetic nomenclature has been used for chromosomal rearrangements since the establishment of a G-banding landmark system in 1988 [43]. Genomic coordinates of G-bands have been estimated previously based on band positions on the reference idiogram relative to chromosome length [1]. These estimations were used to determine oligo-banding equivalences. Centromere positions relative to oligo-banding probes were also used to validate these equivalences. Among the 18 autosome pairs, only SSC6 and SSC7 contained mismatching genomic addresses. In both cases, our centromere positions were before the second probes, in contrast with previously estimated locations distal to these probes [1]. Based on repetitive sequence analysis of the Sscrofa 11.1 genome, centromere placement has been proposed for several chromosomes [44]. Sequence analysis of the SSC6 and SSC7 centromeres gave results that are consistent with the probe-based centromere positions determined in this study (See Additional file 6: Table S3).

Another mismatch with the G-banding estimated genomic positions was detected on SSCY. In this case, the centromere location was previously estimated to occur between our two oligo-banding probes [1]. However, in situ experiments showed that both these probes hybridized on the p arm. The SSCY q arm consists mainly of repeated sequences [45]. Scaffolding in repetitive regions is a daunting task even with current bioinformatic technologies [46], and the SSCY q arm has yet to be assembled. Oligo-banding probe design was based on the available SSCY p arm assembly only. The centromere-probe positions determined in this study appeared to be correct. Thus, the inconsistency between the SSCY probe positions described here and the G-band-estimated positions [1] might be due to the use of the p arm length as the whole SSCY length.

In this study, we have demonstrated the utility of oligo-banding for investigating chromosomal abnormalities by detecting and confirming four translocations and an unbalanced abnormality among boars from a Canadian breeding nucleus. Unbalanced structural abnormalities are often associated with potential lethality, since they result in the gain or loss of a copy of the included genes [3, 47]. Malformations in offspring that develop to term have been reported [17, 48, 49]. In our study, the pig that carried additional material on SSC10 had a normal phenotype, and the only difference with other boars was that it failed the semen conservation tests. The extra material that was detected by oligo-banding was on the p arm of SSC10 between the first probe and the centromere, and included a red-labelled fragment. Previous studies have revealed an active nucleolar organizer region (NOR) in this part of SSC10 [43, 50–52]. Such regions contain repeated ribosomal gene clusters (rDNA) in tandem [53]. Considerable interspecies and intraspecies variability within rDNA has been reported previously [52–54], mainly in terms of cluster abundance and location [55, 56]. Studies in *Lolium spp*. and *Mus* have shown a positive correlation between rDNA clusters and chromosomal breakpoint regions [56, 57]. Thus, this addition of chromosomal material to SSC10 could have occurred during chromosome repair. However, since no fragile site/hotspot has been reported in this region [1, 58] and only one known translocation appears to have occurred on SSC10p11 [11], this abnormality might be a coincidence.

Of the four translocations studied, the translocation between SSC3 and SSC9 has never been reported. The three others have been reported either identically, such as rcp(12;14)(q13;q21), or with different G-band breakpoints [1, 6, 11, 59, 60]. It has been reported that translocation breakpoints occur preferentially within negative G-bands or euchromatin-rich regions [1, 61]. Moreover, Giemsa-negative bands containing a fragile site are reportedly the most prone to translocations [1]. In our study, all breakpoints occurred within G-negative bands, except on SSC7, which was found to break very close to or within the centromere. Among the hundreds of chromosomal rearrangements reported over the past 50 years, translocations between SSC12 and SSC14 are among the most common ones, and both chromosomes have been associated with the highest translocation frequencies of the pig karyotype [1]. This could explain the higher frequency of SSC12-SSC14 translocations within the studied breeding nucleus. The SSC12q13 and SSC14q21 breakpoints that we have described here have also been associated with potential hotspots, and the Giemsa-negative SSC14q21 band has been reported to contain a fragile site [1, 58].

In spite of the large number of reciprocal translocations reported to date, transmission studies of chromosomal abnormalities in pigs remain scarce and most of them have been done using BAC probes [62, 63]. Oligo-banding probes hybridize efficiently on sperm DNA. However, the complexity of the signals due to the numerous possibilities of chromosome segregation makes interpretation challenging. We observed that the state of chromatin decondensation has an impact on signal quality. It has been previously reported that over-decondensed heads can lead to duplicated or unclear signals [64–67]. The main explanation for multiple signals in decondensed spermatozoa is the use of probes that target repetitive regions or a lack of probe specificity and hence labelling of off-target regions [65, 68]. As many as eight loci have been highlighted from a single probe targeting a repetitive region [68]. In our work, off-target signals were largely avoided by using in silico-designed probes known to target only one specific region of the genome, but still some signal duplication occurred. We hypothesized that multiple signals may result when cells are overtreated prior to SpermFISH, causing DNA fragmentation or diffusion of already fragmented DNA, which spreads signals if they are located within labelled regions. Sperm DNA is ultra-compacted, and it has been suggested that DNA fragmentation is natural in pigs and occurs mainly as double-strand breaks [69]. The aptness of oligo-banding to detect the rearrangement between SSC3 and SSC6 in spermatozoa of the rcp(3;6) carrier remains uncertain. Some segregation patterns were clearly observed, but in many cases the patterns were ambiguous. When banding patterns for two chromosomes involve many probes of the same colour or when an unexpected number of signals is obtained due to different segregation types, pattern recognition is more challenging. To solve this issue, the method might be improved by designing probe-specific forward primers, which is the only substructure that is not associated with a specific function in our design (39mers are genome-specific, reverse primers are chromosome-specific and adapters are colour-specific). With a probe-specific amplification design, chromosome bands could have been chosen specifically in accordance with the chromosome rearrangement, reducing the total number of signals and avoiding multiple colour repetition as seen with specific BAC probes in other studies [62, 63].

Oligo-banding showed advantages over conventional banding methods for karyotyping pigs. It may be less sensitive to environmental influences, treatment conditions and chromosome quality [24, 70–73]. Using coloured banding patterns also required a lower level of expertise as bands are easier to distinguish compared to monochrome bands. However, since fewer bands are labelled, the resolution of oligo-banding is lower than that of conventional methods. Thus, small abnormalities that occur between two bands can be missed. Since oligo-banding offers great flexibility, additional probes can be designed to cover those gaps if more resolution is needed. The minimal distance between probes to be able distinguish them has been estimated at 7 to 8 Mb [36], thus the maximum potential resolution of oligo-banding may be estimated to be within this range, which is close to the G-banding estimated resolution [7]. The detection of small subtelomeric translocations between two chromosomes that share the same colour at chromosomal ends (SSC6 and SSC7 for instance) is another limitation of the current oligo-banding technique. Further development might include the use of more fluorophores to increase colour possibilities or to design close but distinct probes of different colours to generate telomere barcodes. Another solution that could be investigated is to create new composite colours by changing the proportion of each fluorophore in order to generate different spectral signals as for multicolour FISH and spectral karyotyping [74]. Compared to probes that are produced using large genomic fragments (BACs) and can contain repeated elements [75], oligo-based probes are highly specific and take less time to produce than the de novo production of BAC probes [76–78], thanks to informatics tools and massively parallel synthesis. Using BAC probes with sub-telomeric sequence-based karyotyping, pig chromosomal abnormalities have been detected [8, 9, 24]. However, with fluorescence signals only, breakpoint regions, even sometimes chromosomes, could not be determined [9] while this could be achieved using oligo-banding by generating fluorescent signals distributed evenly along the chromosomes. On the economical side, several studies have reported oligo-based FISH as an affordable method [26, 28, 29]. Oligo-banding cost has been estimated at 39.58$ per sample (slide) including consumable material and starting oligo pools (See Additional file 7: Table S4), which represents an attractive cost for livestock karyotyping considering the advantages offered by this method.

## Conclusions

Oligo-banding was shown to be an efficient method of karyotyping pigs by offering specificity and versatility. The binarized probe signal allows automatization of the whole analysis, increasing its convenience. The ability of oligo-banding to highlight abnormalities with less prior cytogenetic expertise represents added value. With the expanding use of genomics and artificial insemination, stud animals have become very valuable, and their genetic material widely disseminated in commercial herds. Convenient cost-effective karyotyping is a profitable strategy to certify the genomic integrity of elite animals before being put into service.

## Supplementary Information

**Additional file 1: Table S1.** Oligonucleotide information. This Excel file contains four spreadsheets containing oligonucleotide libraries 1 to 4 and one giving the conversion between colour ID and the attributed fluorophore. Each library contains a description of the chromosome, probe and colour attributions for each oligo used in this study. The genome-homologous 39mer are also listed with their respective genomic positions. The “Seq_fin” column contains the final 79mer oligo sequences ordered from *Genscript*.

**Additional file 2: Table S2.** Primers and detection oligo sequences. This Excel file contains each primer sequence linked to the 3’ end of their attributed specific fluorophore “handle”, the chromosome-specific reverse primers containing the T7 sequence at the 5’ end, and each detection oligo sequence with its attributed fluorophore at the 3’ end. All sequences were purchased from *IDT DNA* as standard purified oligos except for the detection oligos, which were purchased as HPLC-purified oligos.

**Additional file 3: Figure S1.** Outputted banding patterns of the Random banding developed tool. The Random banding tool takes as inputs a.csv file describing banding patterns associated with each chromosome and a.json file that includes parameters. It then generates a.png image containing banding patterns of each specified chromosome. Based on the.json parameters, separated fluorophore banding patterns (upper part of the figure) or final colour patterns (lower part) can be generated.

**Additional file 4: Figure S2.** First step of the Oligo-Banding plugin. The first step of the oligo-banding plugin is to trace each chromosome with the tracing tool of ImageJ and save them in ROI manager. The tracing represented in Additional file 4 Figure S2 is associated with one of the first chromosome pairs.

**Additional file 5: Figure S3.** Automatic oligo-banding pattern analysis of the rcp(3;6). The “Analyze” tool of the Oligo-Banding plugin [39] analyzes the fluorophore signals of each traced chromosome and generates an output containing ROI manager tracing ID (ROI column), detected fluorophore banding patterns (Signature column), associated chromosome based on pattern identity (Ref column), the number of differences between the detected pattern and the expected one (Err column) and the reference banding pattern of the associated chromosome (Sequence column) (a). The tool also generates plots for each traced chromosome in order to visualize and confirm detected patterns. For instance, the der(3) and der(6) plots of the rcp(3;6) boar are shown in b and c. Based on outputted results table and signal plots, real chromosomal abnormalities and signal detection errors can be distinguished. d After chromosomal abnormality confirmation, images can be generated using basic ImageJ tools. More details on the plugin are available on the associated GitHub page [39].

**Additional file 6: Table S3.** Correspondence between oligo-banding probes and G-bands and their positions on pig chromosomes. Oligo-banding probe positions are specified starting at the first nucleotide of the first oligonucleotide and ending at the last nucleotide of the last oligonucleotide. G-band positions were estimated by Donaldson et al. [1]. The centromere position relative to oligo-banding probes was confirmed on each chromosome pair. Discrepancy with Donaldson et al. [1] was observed on SSC6, SSC7 and Y. However, our centromere positions match those of Warr et al. [44]. When available, the regions closest to our positioning were included in the *Predicted centromere region (start–end*)** columns based on Warr et al. [44] results. A discrepancy was observed also for the SSC17 centromere position as Warr et al. [44] have detected it at the SSC17 end compared to our results.

**Additional file 7: Table S4.** Estimated cost of the oligo-banding method. Economic estimation of the main steps associated with the oligo-banding method which are Oligo pool cost, PCR step, in vitro transcription (IVT) step, reverse transcription (RT) step and hybridization total cost including all steps (Total). Costs are expressed in Canadian dollars (CAD) and for the hybridization of one sample (slide). Consumable material costs for each step are included but salaries and laboratory and microscopy basics are not included as they may change between laboratories.

## Data Availability

All data generated or analysed during this study are included in this published article (and its additional information files).
