# Supplementary figures and images for: Cytogenetic screening of a Canadian swine breeding nucleus using a newly developed karyotyping method named oligo-banding
Source: Genet Sel Evol. 2023 Jul 10;55:47. doi: 10.1186/s12711-023-00819-w (PMC10332092; doi:10.1186/s12711-023-00819-w)

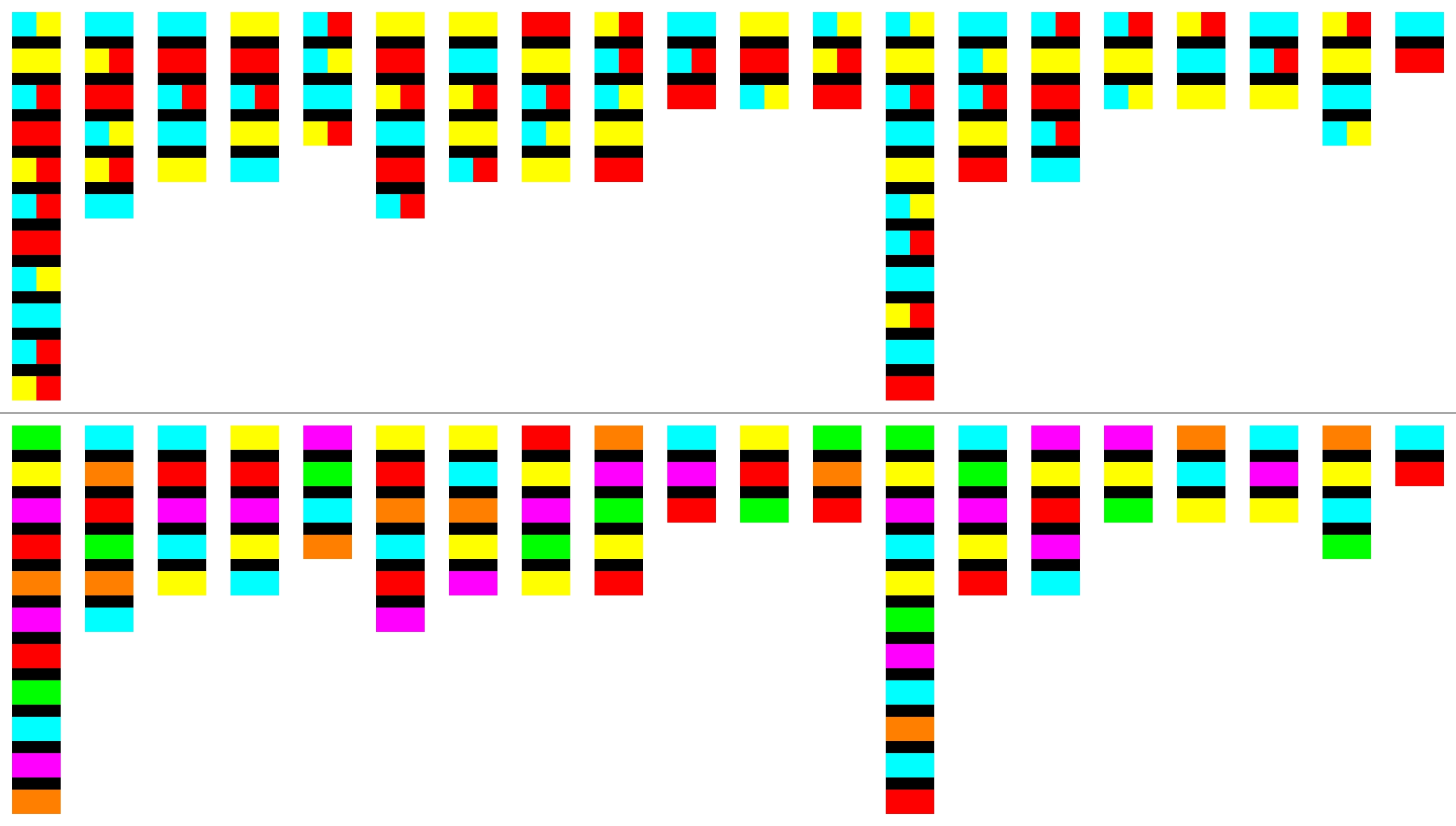

Supplement: Supplementary file 3 — Additional file 3: Figure S1. Outputted banding patterns of the Random banding developed tool. The Random banding tool takes as inputs a.csv file describing banding patterns associated with each chromosome and a.json file that includes parameters. It then generates a.png image containing banding patterns of each specified chromosome. Based on the.json parameters, separated fluorophore banding patterns (upper part of the figure) or final colour patterns (lower part) can be generated. [file 12711_2023_819_MOESM3_ESM.jpg]

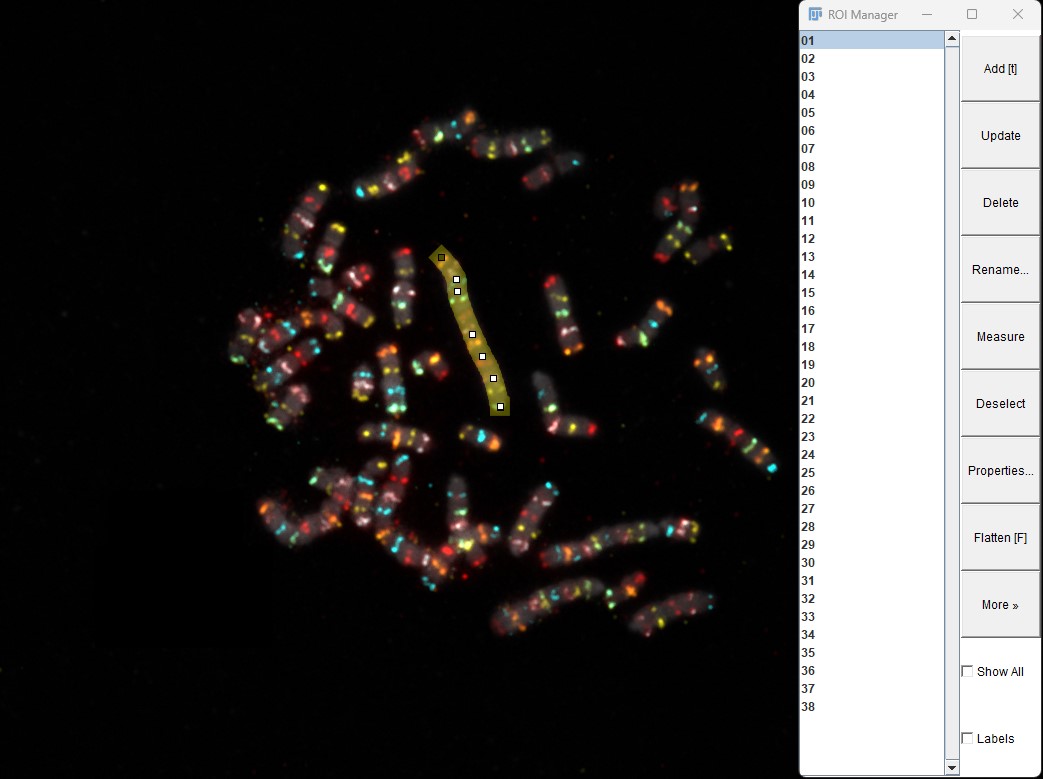

Supplement: Supplementary file 4 — Additional file 4: Figure S2. First step of the Oligo-Banding plugin. The first step of the oligo-banding plugin is to trace each chromosome with the tracing tool of ImageJ and save them in ROI manager. The tracing represented in Additional file 4 Figure S2 is associated with one of the first chromosome pairs. [file 12711_2023_819_MOESM4_ESM.jpg]
