# Supplementary material for: Cytogenetic screening of a Canadian swine breeding nucleus using a newly developed karyotyping method named oligo-banding
Source: Genet Sel Evol. 2023 Jul 10;55:47. doi: 10.1186/s12711-023-00819-w (PMC10332092; doi:10.1186/s12711-023-00819-w)

A

	ROI	Signature	Ref	Err	Sequence
1	01	OWCGRWORWYG	1	0	GYWROWRGCWO
2	02	OWCGRWORWYG	1	0	GYWROWRGCWO
3	03	CORCYOC	2	2	CORGOC
4	04	COGROC	2	0	CORGOC
5	05	YCWRC	3	0	CRWCY
6	06	CRWCOCRW	6	3	YROCRW
7	07	CYWRY	4	0	YRWYC
8	08	CYWRY	4	0	YRWYC
9	09	WGCO	5	0	WGCO
10	10	WGCO	5	0	WGCO
11	11	WRCORY	6	0	YROCRW
12	12	YY	11	2	YRG
13	13	WYOCY	7	0	YCOYW
14	14	YGOYW	7	1	YCOYW
15	15	RYWGY	8	0	RYWGY
16	16	RYWGY	8	0	RYWGY
17	17	OWGYR	9	0	OWGYR
18	18	RYGWO	9	0	OWGYR
19	19	RWC	10	0	CWR
20	20	CWR	10	0	CWR
21	21	GRY	11	0	YRG
22	22	GRY	11	0	YRG
23	23	GOR	12	0	GOR
24	24	GOR	12	0	GOR
25	25	RCOCWGCRYG	13	2	GYWCYGWCOCR
26	26	RCORGCRYG	13	4	GYWCYGWCOCR
27	27	RXWGW	8	2	RYWGY
28	28	CGWYR	14	0	CGWYR
29	29	WYRWC	15	0	WYRWC
30	30	WYRWC	15	0	WYRWC
31	31	GYW	16	0	WYG
32	32	GYW	16	0	WYG
33	33	OCY	17	0	OCY
34	34	YCO	17	0	OCY
35	35	YWC	18	0	CWY
36	36	YWC	18	0	CWY
37	37	GCYO	19	0	OYCG
38	38	RC	20	0	CR

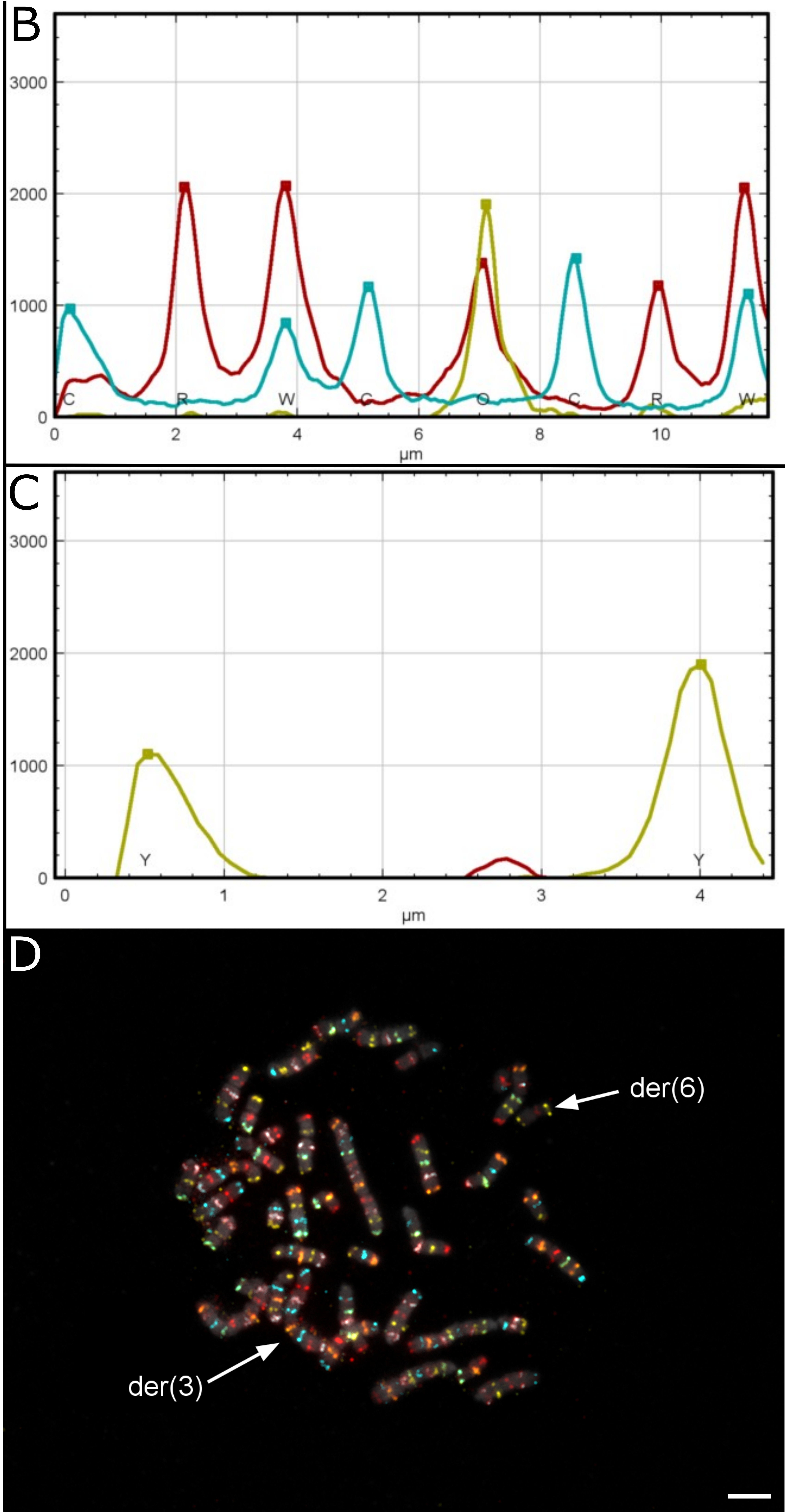

Supplement: Supplementary file 5 — Additional file 5: Figure S3. Automatic oligo-banding pattern analysis of the rcp(3;6). The “Analyze” tool of the Oligo-Banding plugin [39] analyzes the fluorophore signals of each traced chromosome and generates an output containing ROI manager tracing ID (ROI column), detected fluorophore banding patterns (Signature column), associated chromosome based on pattern identity (Ref column), the number of differences between the detected pattern and the expected one (Err column) and the reference banding pattern of the associated chromosome (Sequence column) (a). The tool also generates plots for each traced chromosome in order to visualize and confirm detected patterns. For instance, the der(3) and der(6) plots of the rcp(3;6) boar are shown in b and c. Based on outputted results table and signal plots, real chromosomal abnormalities and signal detection errors can be distinguished. d After chromosomal abnormality confirmation, images can be generated using basic ImageJ tools. More details on the plugin are available on the associated GitHub page [39]. [file 12711_2023_819_MOESM5_ESM.pdf]
